# Association among dysmenorrhea and activity limitation and participation restrictions in adult women: a cross-sectional study, Brazil -2017

**DOI:** 10.1186/s13690-021-00721-1

**Published:** 2021-11-10

**Authors:** Luana Brito dos Santos, Caroline Wanderley Souto Ferreira, Caroline Gomes Gonçalves, Mikaela Aparecida de Oliveira Xavier, Jardelina Hermecina Dantas, Isabelle Ribeiro Barbosa, Saionara Maria Aires da Câmara, Diego Dantas

**Affiliations:** 1 Universidade Federal do Rio Grande do Norte, Programa de Pós-Graduação em Ciências da Reabilitação, Rio Grande do Norte Santa Cruz, Brazil; 2 Universidade Federal de Pernambuco, Programa de Pós-graduação em Fisioterapia, Pernambuco Recife, Brazil

**Keywords:** Pelvic pain, Disability evaluation, Women’s health, International classification of functioning, disability and health

## Abstract

**Background:**

The present study aims to analyse if dysmenorrhea is associated with activity limitations and participation restrictions in experencied by adult women.

**Methods:**

This is a cross-sectional study carried out with adult women recruited from primary health services in Brazil between 2016 and 2017. Dysmenorrhea was measured via a self-report and the activity limitation and participation restrictions was assessed by the World Health Disability Assessment Schedule (WHODAS2.0). Pain and level of physical activity were assessed, respectively, by the numerical pain rating scale and International Physical Activity Questionnaire (IPAQ), short version. Associations between groups, dysmenorrhea and without dysmenorrhea (WD), were analysed by the median difference, Mann Whitney test, effect size measures and 95 % confidence interval. Statistical significance was *p* <0.05.

**Results:**

The prevalence of dysmenorrhea was 54 %. The pain lasted an average of three days or more (66 %). The average pain intensity, in the crisis period, was 6.1 ± 2.6, women who presented severe pain complaints were associated with considerable difficulties in their domains of mobility (*p* = 0.003; 2 = 0.115), participation (*p* = 0.030; 2 = 0.063) and total score (*p* = 0.012; 2 = 0.086), with a moderate effect size for all variables.

**Conclusions:**

Severe dysmenorrhea was associated with higher scores of disabilities assessed by the WHODAS 2.0, especially in mobility, and participation domains.

## Background

Dysmenorrhea is defined as colic pain in the lower abdomen, which occurs with menstruation [1]. It is an important cause of morbidity, being the most common gynaecological complaint amongst women [2]. Although it is more frequent among young women, it can affect women in any age group [3].

Dysmenorrhea is one of the most common causes of absenteeism at school and work, negatively affecting academic commitment and productivity. In addition, it affects daily activities, sexual activities and quality of life [4, 5]. All these factors negatively affect the women’s’ well-being [6] and functioning [7].

Functioning is a generic term, used to describe the positive interaction between the function and structure components of the human body, and its activities and participation, from a biopsychosocial and holistic point of view, whereas disability would be the antonym of functioning and represents negative aspects of this relationship [8]. In 2001, the World Health Organization (WHO) published The International Classification of Functioning, Disability and Health (ICF), being a model for understanding human functioning that encompasses biomedical, social, personal, and environmental factors [9].

The ICF offers a broad classification system, with a high complexity, that requires a long period of time to be spent with each patient. Because of this, new questionnaires, based on the ICF, were developed to optimize its operation [10]. For these reasons, the WHO developed the “World Health Organization Disability Assessment Schedule 2.0’’ (WHODAS 2.0), which is a generic assessment tool that provides a standardized method of measuring functioning and disability in a cross-cultural way. It was developed from a comprehensive set of ICF items, and it evaluates the difficulties that a person has experienced in social participation and activities of daily living, in the last 30 days, and can be used in the general population or in groups with specific health conditions [11].

Some studies, using WHODAS, of disability in women, have been reported in the following areas: oncology [12–15], obstetrics [16–18], urology [19, 20] and sexual function [21]. These studies underscore the importance of understanding women’s health contexts using the ICF; as well as, understand the impact of women’s health problems on their activities and participation, through a comprehensive and standardized instrument.

Despite this relevance, there are no studies that assess the functioning or disability related to dysmenorrhea using the WHODAS 2.0 or ICF taxonomy. In addition, studies that aimed to assess the impact of dysmenorrhea on the quality of life, or in the daily lives of women were restricted to adolescents or young adults [22].

Accordingly, the aim of this study was to analyse whether dysmenorrhea is associated with disability in the performance of activities and participation in adult women.

## Methods

### Study design

This is a cross-sectional study held in the city of Santa Cruz, state of Rio Grande do Norte, in the Northeast of Brazil, between 2016 and 2017. The research protocol was submitted and approved by the Research Ethics Committee of Federal University of Rio Grande do Norte under number: 49237315.9.0000.5568, and was conducted in line with the terms of the Declaration of Helsinki. All participants gave their informed consent for inclusion before they participated in the study.

The sample size was determined using the G * Power software (Düsseldorf, Germany), considering a significance level of 0.05, power of 0.80 to obtain an effect size of 0.5 (moderate) between the variables: dysmenorrhea and disability. Based on these criteria, at least 134 women were required.

In this study, the following categories of women were included: women aged between 19 and 39 years, non-pregnant, who have menstruated in the past 3 months, without endometriosis or chronic pelvic pain diagnosis, and those who accepted to participate in the study after knowing the objectives. Participants with cognitive deficits who compromised the understanding of the questionnaire would have also been excluded; however, there were not any such cases.

Women were recruited and interviewed at the six primary health care services in the city. These services serve all adult women and offer actions to promote health, prevent and treat diseases compatible with primary health care. Women were invited to take part in the study while they were waiting for regular appointments with clinicians, were waiting to join to health education group or accompanied family members. The interviews took place in a quiet and private room, and they were carried out by previously trained interviewers.

### Outcomes

The functioning was measured using a version of 36-item interviewer-administered version from the World Health Disability Assessment Schedule (WHODAS2.0), adapted and validated for the Brazilian population [23]. It is divided into 6 domains: cognition; mobility; self-care; getting along; life activities and participation in society, and reflects the ICF in each item. The 36 questions offer a 5-point Likert scale answer, in which 1 indicates without any difficulty and 5 extreme difficulty or inability to do the activity, in situations experienced in the last 30 days. The complex scoring method was used to calculate the total score and a score for each of the six domains, ranging from 0 (no disability) to 100 (full disability) [11].

Dysmenorrhea was assessed using a self-report by participants with a history of colic-type pain in the lower abdomen, associated with menstruation, in the last three months [1]. The average duration of dysmenorrhea was evaluated in days, and the average pain intensity in a crisis period, using the Numerical Pain Scale (NPS) from 0 to 10. For the purpose of statistical analysis, the average painful intensity was dichotomized into severe pain, with an intensity ranging from 7 to 10; and mild to moderate pain, comprising pain with an intensity from 1 to 6 on the NPS [24].

A questionnaire designed specifically for this research was used to characterize the sample, addressing socio-demographic data and gynaecological characteristics to extract information about: age, number of children, level of education, marital status, family income, age at menarche, average duration of the menstrual cycle and use of contraceptive methods.

The level of physical activity was assessed using the International Physical Activity Questionnaire - IPAQ, short version, which had previously been translated and validated for the Brazilian population [25]. The IPAQ quantifies energy expenditure in metabolic equivalents of Task (METs) - minute/week with physical activity.

### Data analysis

Data analysis was performed using the Statistical Package for the Social Science SPSS® software version 20.0. To characterize the sample, the data was presented in absolute and relative frequencies, averages and standard deviations; as well as, median and interquartile ranges. The distribution of data was evaluated by the Kolmogorov-Smirnov test. Parametric variables were compared using the T-test for unpaired data and categorical variables by Chi-square test. The variables resulting from WHODAS 2.0 (total and domains) presented a non-parametric distribution. For this reason, the Mann-Whitney U test was used to compare the impact of dysmenorrhea and its characteristics on the WHODAS 2.0 score. It was considered statistically significant when *p* <0.05. Cronbach’s alpha assess the internal consistent of WHODAS 2.0 items in women with dysmenorrhea. The effect size was calculated by eta square (η2) and the value of η2 interpreted as small (≥0.01), medium (≥0.06) or high (≥0.14) [26]. The Hodges-Lehman estimator provided the difference in the median and the 95 % confidence interval between groups.

## Results

Out of 136 women that were included in the study, 73 (54 %) had dysmenorrhea. Table 1 shows the comparison between the groups of women with dysmenorrhea and without dysmenorrhea (WD), for the variables: age, number of children, education, marital status, income, use of contraceptive method, age at menarche, duration of menstrual cycle and METs spent on physical activity. There were no significant differences in the results for each of these variables analysed between the two groups.

**Table 1:** Characterization of the sample and comparison between women with and without dysmenorrhea (Brazil - 2017, *n* = 136)

Variables	D (*n* = 73) Mean±SD / n (%)	WD (*n* = 63) Mean±SD / n (%)	p
**Age (years)**	28.4±6.7	27.7±5.9	0.491
**Number of children**	1.62±1.48	1.48±1.00	0.513
**Level of Education**			
Up to elementary school	34(47)	24 (38)	0.414
High school or higher	39 (53)	39 (62)	
**Marital status**			
Without partner	37 (51)	30 (47)	0.827
With partner	36 (49)	33 (53)	
**Family income**^a^			
Up to 1 Brazilian minimum wage	59 (81)	49 (78)	0.495
>1 Brazilian minimum wage	14 (19)	14 (22)	
**Use of contraceptive method**			
Yes	37 (51)	34 (54)	0.765
No	36 (49)	29 (46)	
**Age of menarche (years)**	12.81±1.45	12.71±1.36	0.686
**Average menstrual cycle duration (days)**	29.22±10.10	29.18±11.81	0.986
**MET (MET-min/week)**	8424.1±36,152.9	5558.0±9,880.0	0.541

*D* Dysmenorrhea, *WD* Without Dysmenorrhea, *SD* Standard Deviation, *MET* Metabolic Equivalent of Task, *min/week* minute/week

^a^The value of the Brazilian minimum wage, in 2016, was $ 270.76

Table 2 shows a comparison between the groups regarding WHODAS scores. There were no significant differences between the groups. The items of WHODAS 2.0 showed a good internal consistency (α = 0.868).

**Table 2:** WHODAS scores’ comparison between groups (Brazil, 2017)

WHODAS	D (*n* = 73) Median(IR)	WD (*n* = 63) Median(IR)	Median difference	CI 95 %	p	η^2^
**Total score**	8.69 (22.28)	16.85 (14.87)	-1.087	-4.35 to 3.261	0.782	0.001
**Cognition**	15.00 (25.00)	15.00 (15.00)	0.00	-5.00 to 5.00	0.738	0.001
**Mobility**	12.50 (31.25)	12.50 (31.25)	0.00	-6.25 to 6.25	0.945	0.000
**Self-care**	0.00 (10.00)	0.00 (0.00)	0.00	0.00 to 0.00	0.627	0.001
**Getting along**	8.33 (16.67)	8.33 (25.00)	0.00	- 8.33 to 0.00	0.175	0.012
**Life activities**	10.00 (30.00)	10.00 (30.00)	0.00	0.00 to 0.00	0.955	0.000
**Participation**	16.67 (37.50)	12.50 (20.83)	0.00	-4.16 to 8.33	0.651	0.001

Note: Higher WHODAS values indicate greater disability

The median difference was calculated by the Hodges-Lehman estimator; η2: Eta squared

*D* Dysmenorrhea, *WD* Without Dysmenorrhea, *IR* Interquartile Range, *CI 95%* Confidence Interval 95%, *pp*-value calculated by the Mann-Whitney test

The average pain intensity, in the crisis period, was 6.10 ± 2.57 and 44 % reported suffering from severe pain. Women with severe pain had more difficulties in the domains mobility (*p* = 0.003) and participation (*p* = 0.030), as well as in the total WHODAS score. All associations had a moderate effect size (Table 3).

**Table 3:** Effect of pain intensity on WHODAS 2.0 scores among women with dysmenorrhea (Brazil – 2017, *n* = 73)

WHODAS	Severe pain (*n* = 32) Median(IR)	MMP (*n* = 41) Median(IR)	Median difference	CI 95 %	p	η^2^
**Total score**	20.00(25.00)	10.00(30.00)	7.61	2.17 to 11.96	0.012	0.086
**Cognition**	20.00(25.00)	10.00(30.00)	5.00	0.00 to 15.00	0.079	0.041
**Mobility**	18.7(31.25)	6.25(25.00)	12.50	6.25 to 18.75	0.003	0.115
**Self-care**	0.00(10.00)	0.00(10.00)	0.00	0.00 to 0.00	0.874	0
**Getting along**	8.33(22.92)	8.33(16.67)	0.00	0.00 to 8.33	0.608	0.003
**Life activities**	10.00(30.00)	10.00(20.00)	0.00	0.00 to 10.00	0.679	0.002
**Participation**	20.83(25.00)	8.33(27.08)	8.33	0.00 to 16.66	0.030	0.063

Note: Higher WHODAS values indicate greater disability

The median difference was calculated by the Hodges-Lehman estimator; η2: Eta squared

*MMP* Mild to Moderate pain, *IR* Interquartile Range, *CI 95 %* Confidence Interval 95 %, *p* *p*-value calculated by the Mann-Whitney test

The majority of women (66 %) stated that the pain persisted for three or more days. However, the length of time of the pain did not influence the WHODAS 2.0 scores (Table 4).

**Table 4:** Comparison of dysmenorrhea crisis length in relation to WHODAS 2.0 domains (Brazil – 2017, *n* = 73)

	Duration of pain					
WHODAS	≥ 3 days (*n* = 32) Median(IR)	1 or 2 days (*n* = 41) Median(IR)	Median difference	CI 95 %	p	η ^2^
**Total score**	18.48(21.74)	13.59(17.39)	3.26	-3.26 to 8.70	0.308	0.035
**Cognition**	15.00(27.50)	15.00(25.00)	0.00	-5.00 to 10.00	0.761	0.001
**Mobility**	12.50(31.25)	12.50(31.25)	0.00	-6.25 to 6.25	0.661	0.003
**Self-care**	0.00(10.00)	0.00(10.00)	0.00	0.00 to 0.00	0.796	0.001
**Getting along**	8.33(20.83)	4.16(16.67)	0.00	0.00 to 8.33	0.431	0.008
**Life activities**	10.00(20.00)	0.00(40.00)	0.00	-10.00 to 10.00	0.961	0.00
**Participation**	20.83(43.75)	12.50(28.13)	4.17	-4.16 to 20.83	0.213	0.021

Note: Higher WHODAS values indicate greater disability

The median difference was calculated by the Hodges-Lehman estimator; η2: Eta squared

*IR* Interquartile Range, *CI 95%* Confidence Interval 95%, *pp*-value calculated by the Mann-Whitney test

## Discussion

Based on the ICF taxonomy, disability is the opposite of functioning and it is related to problems or difficulties in the ICF components and in their respective domains [8]. In the present study, no association was found between dysmenorrhea and the duration of the crisis with the WHODAS score and its domains. However, the intensity of the pain complaint was associated with disability. Furthermore, women with severe pain had higher scores for general disability, and for the mobility and participation domains.

Pain intensity proved to be an important parameter to verify the associations between dysmenorrhea and the disability of adult women. Comparing the pain intensity observed in adult women with other age groups, it is observed that the percentage of adult women who reported severe pain, although considerable (44 %), is lower than with data previously reported in the literature [3] for studies of young adult women, 80.3 % [27] and adolescents 52.5 % [6].

Pain intensity is a multidimensional variable, that involves numerous criteria such as culture, lifestyle, genetics, and the degree of social and personal stress [3, 22, 28–30]. Therefore, the more intense this experience is, the larger the repercussions are on the lives of the women who face it. In addition to the effects on mobility, getting along and participation, women with dysmenorrhea have greater difficulty in cognitive activities, concentration, memory, problem solving, learning, communication and reasoning activities [31].

Women in severe pain had more difficulties for activities involving mobility, such as standing, moving around the house, leaving the house and walking long distances [31]. In addition, the pain intensity was associated with considerable participation restrictions, which refer to social dimensions, such as activities, barriers and obstacles in the environment and problems with other issues such as maintaining personal dignity, including contextual factors (personal and environmental) [31].

In the present study, women with severe dysmenorrhea had worse performance in activities involving interaction and interpersonal relationships. According to the WHO, the WHODAS 2.0 getting along domain encompasses interactions with other people, that can be close to them or not, and difficulties that can be found with this domain of life due to health conditions [31]. Our findings corroborate with a previous study carried out with adolescent women [28], found that dysmenorrhea interfered in the relationship with friends/colleagues of 17.7 % of the adolescents interviewed.

Besides that, our data points to a negative relationship between dysmenorrhea and social participation. This finding shows that negative impacts previously documented in adolescents and young adult women, can remain in adult women who suffer from dysmenorrhea and have severe pain. Current evidence supports the association between dysmenorrhea and losses in academic performance and productivity at work [22, 28, 32–36] and negative performance in the practice of sports and leisure activities [28, 37].

The findings from this study, together with the literature review, show how dysmenorrhea negatively impacts women’s lives. In this sense, it is necessary for health professionals to monitor the health needs of women, with dysmenorrhea, from a biopsychosocial perspective. Many women who have dysmenorrhea do not seek treatment, because they consider pain to be a normal condition of the menstrual cycle, and when they seek health professionals, they present inadequate knowledge about pain management, which can translate into negative attitudes about dysmenorrhea which discourage women to seek appropriate treatment [29, 30].

Although there are studies that assess the impact of dysmenorrhea on quality of life [4, 5, 7, 35], in their work and academic activities [22, 28, 32–36], as well as their participation in physical exercise [28, 37], there are no studies that assess functioning with ICF-based instruments. This study is pioneering and used a valid instrument, based on the ICF, to assess and compare disabilities between women with and without dysmenorrhea.

Many studies assess the impact of dysmenorrhea with quality of life [4, 5, 7, 35]. Conceptually, even if the definitions of quality of life and functioning are associated, but instruments used to assess quality of life, assess subjective well-being in different areas of people’s lives, and instruments that measure functioning, such as WHODAS, assess objective performance in a given domain of life, for this reason studies are needed that assess functioning globally and not subjectively [11].

As it was a cross-sectional study, it was not possible to find any cause and effect relationship. In order to confirm the causality of the relationships between the variables described here, it is recommended to develop a new research with longitudinal designs that use WHODAS to assess the impact of dysmenorrhea, as well as the influence of treatments for dysmenorrhea.

In addition, the fact that WHODAS evaluates the limitation of women’s activities in the last 30 days, and not only during the menstrual cycle; as well as, the non-standardization of the women’s cycle period at the time of the assessment, could have underestimated or overestimated women’s disabilities and the level of pain. So, it is suggested that these variables should be controlled in future research.

Despite the pioneering results documenting the disabilities of women with dysmenorrhea through WHODAS 2.0, the data presented here need to be interpreted with caution. Although we interviewed women in all primary health care services in the city, convenience sampling can represent a selection bias, so future studies should include random sampling techniques.

The fact that we did not find an association between the diagnosis of dysmenorrhea and the production of activity limitations may indicate that the chronicity of the condition and other variables would have a more relevant role in the production of disabilities than the diagnosis itself. However, studies with a longitudinal design need to be developed to better investigate these issues, since the limitations of the cross-sectional study, selection and absence of clinical examinations can compromise these findings.

## Conclusions

Dysmenorrhea has a high prevalence in ault and mild-aged women. The pain intensity of dysmenorrhea is the main parameter associated with disability and not the diagnosis itself. Thus, adult women with severe pain crises have higher disability scores in the WHODAS total score, and in the domains of mobility and participation.

These results show the importance of planning and executing strategies for comprehensive health care for these women, developing treatment and monitoring programs based on functioning.

## Data Availability

The datasets used and/or analysed during the current study available from the corresponding author on reasonable request.

## Abbreviations

ICF: International classification of functioning, disability and health
IPAQ: International physical activity questionnaire
METs: Metabolic equivalents of task
NPS: Numerical pain scale
WD: Without dysmenorrhea
WHO: World Health Organization
WHODAS 2.0: World Health Organization disability assessment schedule 2.0
